# Enhanced cuticular penetration as the mechanism for synergy of insecticidal constituents of rosemary essential oil in *Trichoplusia ni*

**DOI:** 10.1038/srep12690

**Published:** 2015-07-30

**Authors:** Jun-Hyung Tak, Murray B. Isman

**Affiliations:** 1 Faculty of Land and Food Systems, University of British Columbia, Vancouver, British Columbia, V6T 1Z4, Canada

## Abstract

Synergistic interactions between constituents of essential oils have been reported for several areas of research. In the present study, mechanisms that could explain the synergistic action of the two major insecticidal constituents of rosemary oil, 1,8-cineole and camphor against the cabbage looper, *Trichoplusia ni* were investigated. 1,8-Cineole was more toxic than camphor when applied topically to larvae, and when coadministered in their ratio naturally occurring in rosemary oil, the binary mixture was synergistic. However, when injected directly into larvae, camphor was more toxic than 1,8-cineole. GC-MS analyses showed that penetration of topically-applied camphor was significantly enhanced when it was mixed with 1,8-cineole in the natural ratio. A bioassay combining injection and topical application methods confirmed the increased penetration of both compounds when mixed, showing the same bioactivity as seen for higher amounts applied individually. Lowered surface tension as well as increased solubility of camphor by 1,8-cineole, along with the interaction between 1,8-cineole and the lipid layer of the insect’s cuticle may explain the enhanced penetration of camphor. Because of the similarities in biological function of animal and microbial membranes, our finding has potential for application in other fields of study.

Not unlike the pharmaceutical activity of drugs in animals and humans, insecticidal activity is the result of a series of complex actions and counteractions between a toxicant and an insect’s tissues. These complex dynamics of toxicity can be simplified into three categories - penetration, activation (= target site interaction) and detoxification^1^. For insecticidal activity to ensue, the toxicant must penetrate the insect’s integument as well as the membranes of target organs, but some portion of it may be metabolized and neutralized by evolutionally-developed insect defense mechanisms before reaching the target site(s). Ultimately, the amount of toxicant reaching the target site will determine the toxicity of the insecticide.

To show enhanced toxicity of a combination of toxicants (i.e., synergy), at least one of these categories needs to be affected, e.g., penetration is increased, the accumulated compound shows a higher level of activation, or less of the active compound is detoxified. The study of insecticide synergy has mostly focused on biochemical aspects of activation or detoxification^2^,^3^,^4^,^5^, whereas enhanced penetration as a possible mechanism of synergy has been relatively less explored. In the present study, our main question was whether there is increased penetration of a specific combination of rosemary essential oil constituents, and if this could account for their observed synergistic action. In addition, possible mechanisms for enhanced penetration were examined.

## Results

### Contributions of the major constituents to toxicity of rosemary oil

**Figure 1:**
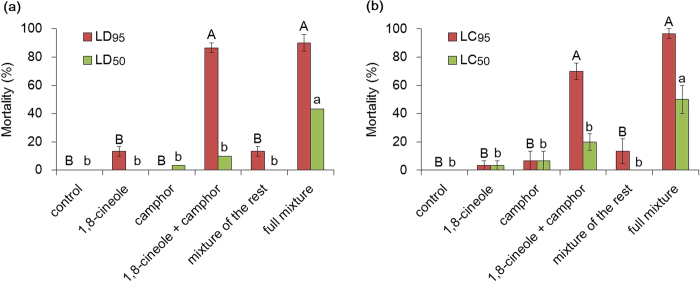
Insecticidal activities of individual major constituents of rosemary oil and their binary mixture via (**a**) topical and (**b**) fumigation assay methods. Error bars represent the standard error of the mean of three replicates of 10 larvae each. Bars with the same letter indicate no significant differences (Tukey HSD test, *P* < 0.05); upper case letters refer to the higher doses (LD_95_ or LC_95_), lower case letters to the lower doses (LD_50_ or LC_50_).

The individual contributions of the two most abundant compounds, 1,8-cineole and camphor in rosemary oil and their combined effect to the toxicity of the oil were examined (Fig. 1). In both topical and fumigant bioassays, an artificial mixture consisting of ten major constituents of rosemary oil (>1% in concentration) reproduced the bioactivity of natural rosemary oil. While both 1,8-cineole and camphor showed limited toxicity when individually applied, their binary mixture showed significantly increased toxicity, comparable to that of the full mixture at the LD_95_ and LC_95_ doses (topical, *P* = 0.70 / fumigant, *P* = 0.06), indicating a synergistic interaction of the compounds in both application methods (χ^2^ = 4.21 and 4.13, respectively). In lower concentrations (LD_50_ and LC_50_ levels), although there were slight increases in toxicity of the binary mixture, they did not reach those of the artificial full mixture (*P* = 0.03 and 0.04, respectively).

### Analysis of the uptake of 1,8-cineole and camphor through the insect cuticle

**Figure 2:**
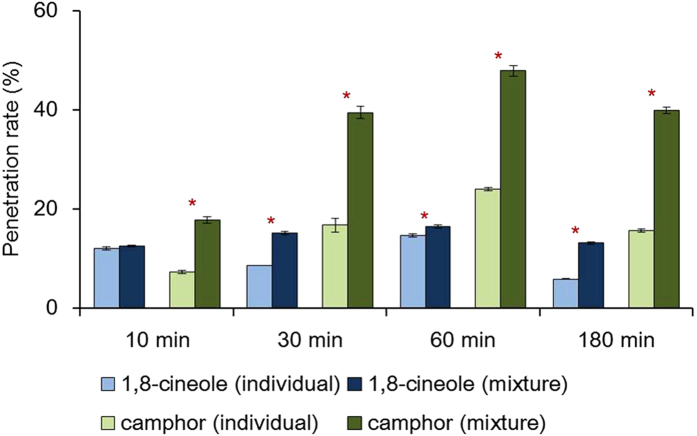
Penetration rate of the compounds when applied individually or when mixed in 3rd instar larvae of *Trichoplusia ni*. Error bars indicate standard deviations (n = 3), and asterisks indicate statistical differences between individual and mixed applications within each time of observation (*P* < 0.05). Both compounds showed increased penetration rates when they were mixed (*P* < 0.05, except 1,8-cineole at 10 min), but the increase rate for camphor (15.9 to 36.2%, on average) was much greater than that for 1,8-cineole (10.3 to 14.3%).

The residual (unpenetrated) and penetrated amounts of 1,8-cineole and camphor in larvae of the cabbage looper (*Trichoplusia ni*) were analyzed by GC-MS. Quantities in larval rinses (representing the amount of material that failed to penetrate the cuticle) showed a decreasing pattern over time, whereas internal quantities (extracted from homogenates of rinsed whole bodies) increased for 1 h after application, but decreased at 3 h post-treatment (See Supplementary Table S1 online). When the two compounds were mixed, there was a substantial increase in penetration of camphor to more than double the amount for its individual application (*P* < 0.05, from 15.9% to 36.2%, on average, Fig. 2). The average penetration rate of 1,8-cineole was also significantly increased when mixed with camphor except the observation at 10 min (*P* < 0.05, from 10.3% to 14.3%, on average), but the rate of increase was lower. The recovery rate of camphor was higher than that of 1,8-cineole in all analyses.

### Insecticidal activity of the compounds via injection assay

The toxicities of each compound by injection were examined and compared to those from topical administration (Table 1). In a previous report, 1,8-cineole had a lower LD_50_ value (229.6 μg/insect) than camphor (471.3 μg/insect) against 3rd instar larvae of the cabbage looper via topical application^6^. However, when injected into 5th instar larvae, camphor had a significantly lower LD_50_ than 1,8-cineole, with a toxicity ratio of 81.3, compared to 1,8-cineole with a ratio of 15.6. Given the observation of enhanced penetration of camphor, higher internal toxicity suggests camphor as being more responsible for the insecticidal activity of the 1,8-cineole + camphor binary mixture.

### Comparative toxicity in mixed application assays

**Figure 3:**
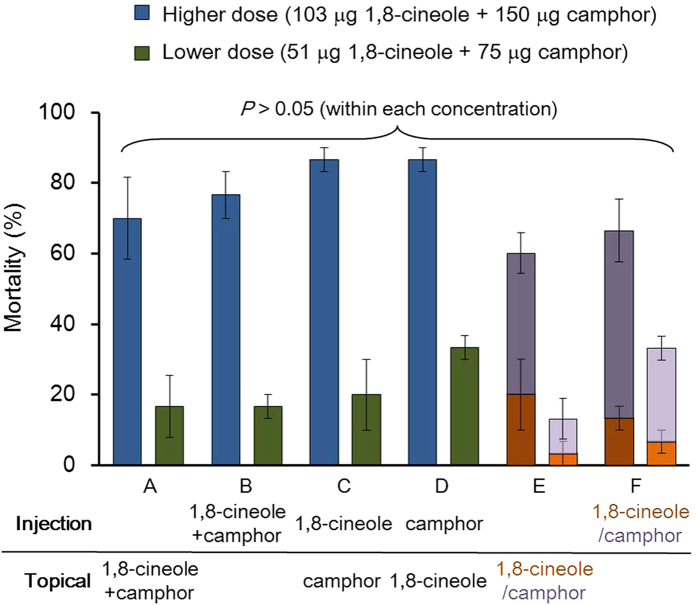
Mortalities caused by 1,8-cineole and camphor in a mixed application assay against 5th instar larvae of the cabbage looper. Error bars indicate standard errors of the mean (n = 3). Each application was conducted by (**A**) topical administration of the mixture, (**B**) injection of both compounds as a mixture, (**C** and **D**) injection of one compound and topical application of the other one, or (**E** and **F**) topical application or injection of individual compounds. **E** and **F** represent the sum of mortalities of individual topical applications or injections of the two compounds.

Mortalities resulting from the individual compounds and the mixture of 1,8-cineole and camphor in a mixed application assay using 5th instar larvae of the cabbage looper were examined (Fig. 3). All four permutations of topical administration (A) and injection (B) of the mixture, and topical + injection at theoretical (topical) or actual (by injection) penetrated doses (C and D) showed no difference in toxicity at either concentration level (higher, *P* = 0.33 and lower, *P* = 0.35), nor did they differ from the sum of the mortalities from individual applications of the compounds at either concentration (A through F, *P* = 0.08 and 0.23, respectively).

### Surface tension and contact angle measurement

The contact angles of acetonic solutions of selected compounds and their combinations on a layer of beeswax were measured as well as their surface tensions without the solvent (Table 2). Although differences between the test groups were statistically different (*P* < 0.05), the magnitude of difference was slight and probably not biologically meaningful. On the other hand, the camphor solution displayed a significantly higher contact angle on beeswax than did the other materials (*P* < 0.05). 1,8-Cineole solution had the lowest contact angle, and the mixed solution of 1,8-cineole and camphor had a relatively similar angle to that of rosemary oil. When camphor was mixed with all other constituents of rosemary oil except 1,8-cineole, it also showed a similar contact angle to that of 1,8-cineole + camphor and rosemary oil (*P* > 0.05).

## Discussion

Plant essential oils have been a subject of research by many disciplines because of their wide range of bioactivities including antimicrobial, insecticidal, therapeutic and medicinal effects. The activity of an essential oil will sometimes depend on that of its major constituent, but the opposite is also possible, i.e., overall activity of the oil cannot be explained by the sum of the activities of individual constituents, indicating synergistic or antagonistic effects. Recent research on *Lippia sidoides* oil showed that although thymol constituted 85% of the oil, the intact oil showed antimicrobial activity superior to that of pure thymol^7^. Similar reports of partial and incomplete activity of individual major constituents of essential oils on microorganisms and insects suggest synergy with minor constituents of essential oils^8^,^9^,^10^,^11^,^12^. Synergy can be also found between essential oils^13^ or terpene compounds^14^,^15^, even in their interactions with synthetic antimicrobials^16^, insecticides^5^, or other natural organic and inorganic materials such as amphotericin B^17^, *Bacillus thuringiensis*^18^, diatomaceous earth^19^ and vanillin^20^,^21^.

1,8-Cineole, the major constituent of rosemary (*Rosmarinus officinale*) and eucalyptus (*Eucalyptus globus*) oils showed interesting combination effects in several areas. Synergistic bioactivity was reported when it was mixed with aromadendrene^22^, chlorhexidine digluconate^12^, limonene^15^, or other minor constituents of essential oils^8^,^22^. Likewise, the synergistic effect of camphor with other compounds was also reported^23^,^24^, and Pavela^25^ recently reported the notable boosting effect of camphor with other terpenoid compounds. In the present study, 1,8-cineole and camphor showed limited toxicity when individually applied, but binary mixtures were synergistic in both topical and fumigant bioassays on the cabbage looper.

Despite vigorous efforts to understand the mechanism, we still know little about how synergy can be produced. From the pharmaceutical point of view^26^,^27^, the synergy mechanism was suggested as (1) a multi-target effect in which compounds target different sites, (2) pharmacokinetic or physicochemical effects on improved solubility or bioavailability, or (3) interactions of agents with resistance mechanisms.

The rapid action of essential oils against some insect species is indicative of a neurotoxic mode of action, with some evidence of interactions with the neuromodulator octopamine and GABA-gated chloride channels^28^. Also, several essential oils and monoterpenes have been shown to inhibit acetylcholinesterase (AChE)^29^,^30^. Zhukovskaya^31^ found that 1,8-cineole can stimulate the response of pheromone sensitive sensilla of American cockroaches, *Periplaneta americana*, receptor cells of which are modulated by octopamine. In a study of housefly GABA receptors, 1,8-cineole and camphor both showed significant effects on the binding of [^3^H]-TBOB, the picrotoxin binding site of insect GABA receptors, but they showed different response patterns^32^. Studies on AChE showed a species-dependent response as 1,8-cienole had both positive^33^ and negative inhibitions^34^ on insect AChE, but camphor did not show any inhibitory activity^35^,^36^. Moreover, on bovine erythrocyte AChE the mixture of 1,8-cineole and camphor had an antagonistic effect^33^. Although the insecticidal mode of action of the two compounds might differ, they might share the same metabolic fate. 1,8-Cineole was reported to be hydroxylated by the pyrgo beetle, *Paropsisterna tigrina*^37^ and Leichhardt’s grasshopper, *Petasida ephippigera*^38^, and camphor was hydroxylated by the tobacco cutworm, *Spodoptera litura*^39^ when the insects were fed diets containing these compounds.

In terms of penetration of compounds, although the chemical compositions and physical constructions of human (or mammalian) skin and the integument of insects are quite different, they share the same biological functions of protecting the body from xenobiotics and reducing evaporative water loss. The principal barrier to topical drug delivery in humans is the stratum corneum (SC), a complex mixture of lipids embedded in an intercellular matrix of dense dead cells^40^,^41^. On the other hand, the insect integument can be considered a two-phased structure, with lipophilc (epi- and exocuticles containing lipids, lipoprotein and protein) and hydrophilic (endocuticle, a chitin-protein complex) layers^42^. It is generally accepted that hydrocarbon or nonpolar terpenes are considered good penetration enhancers of lipophilic drugs in human skin, whereas polar terpenes provide better enhancement of hydrophilic ones^41^. In particular, 1,8-cineole was proven to be a promising penetration enhancer of several drugs including curcumin^43^, 5-fluorouracil^44^, mefenamic acid^45^, and zidovudine^46^ either *in vivo* or *in vitro*. The penetration enhancing mechanism of 1,8-cineole was suggested to be achieved by making the SC lipids less ordered (i.e., increasing lipid fluidity)^44^,^46^. Insecticide penetration enhancers have been relatively less explored than pharmaceutical ones, but it has been observed that some resistant strains of insects have a thickened cuticle layer that could delay the penetration of insecticides as one resistance mechanism^47^,^48^. We previously reported the pharmacokinetic fate of thymol with different carriers including 1,8-cineole and rosemary oil, but there were no apparent trends related to the synergies observed^49^.

In terms of resistance mechanisms, plant essential oils and terpenoid compounds show some promise in addressing current insecticide resistance problems. Some essential oils can markedly inhibit resistant strains of gram-negative pathogenic bacteria, such as *Klebsiella pneumoniae*^50^ and *Escherichia coli*^51^. Other reports show that although essential oils were less potent than conventional insecticides against susceptible cockroaches, the oils showed consistent toxicity to resistant strains as well, indicating their different mode of action^52^. A recent report by Tong and Bloomquist^5^ showed not only synergistic interactions of essential oils with synthetic pesticides to the yellow fever mosquito, *Aedes aegypti*, but also inhibitory activities on detoxifying enzymes including cytochrome P450 monooxygenases and carboxylesterases, which are well-known to be related to the development of resistance to conventional pesticides.

Since most essential oils are complex mixtures, they themselves might also impede the ability of insects or other organisms to evolve resistance. For example, when pure azadirachtin, the major insecticidal constituent from the Indian neem tree, *Azadiracta indica*, or a refined neem seed extract at the equivalent amount of azadirachtin were repeatedly sprayed on the green peach aphid, *Myzus persicae*, only the pure compound-treated line showed 9-fold resistance to the compound after 40 generations^53^. Diffused selection of resistance by blending with other constituents instead of a single active ingredient can be a useful strategy which is not limited to botanical insecticides. Transgenic broccoli having two different *Bt* toxins showed slowed development of resistance in lepidopteran insects compared to those having a single *Bt* toxin^54^.

In the present study, we found a reversed order of toxicity for camphor and 1,8-cineole between injection and topical assays (Table 1). This enhanced insecticidal activity of camphor when the cuticular barrier was bypassed, combined with the observation of its increased penetration when admixed with 1,8-cineole (Fig. 2) can explain the synergy between the two compounds. This is clear when comparing toxicities of mixtures and individual applications (Fig. 3). When the mixture of the two compounds was injected, the mortality in 5th instar larvae did not differ from the sum of mortalities of individual injected compounds at the same doses of application (B and F, 1,8-cineole: 103 μg/insect, camphor: 150 μg/insect, *P* = 0.90), whereas in topical applications, the mixture of much lesser amounts (A, 1,8-cineole + camphor: 718 + 414 μg/insect) than those of individual topical applications (E, 1,8-cineole: 997 μg/insect, camphor: 943 μg/insect) produced the same degree of toxicity (*P* = 0.90). These comparisons lead us to conclude that the synergy between 1,8-cineole and camphor largely results from the increased penetration of the compounds, especially that of camphor in this particular combination. Mixed applications of calculated doses (C and D) showed the same general toxicities as for the other applications, further confirming the penetration-enhancing effect, and that the injected compounds do not influence penetration of the other compounds that are topically applied. Thus, this effect only can be produced when compounds were topically applied as a mixture.

To understand the penetration-enhancing effect of the mixture, the increased affinity of camphor, the more toxic compound, to the integument of the insect via lowered surface tension might be considered as one possible explanation. The acetonic solution containing 1,8-cineole and camphor in mixture had a relatively lower contact angle on beeswax than a solution containing camphor alone. Since beeswax has a chemical composition similar to the wax layer of the insect integument^55^, we can assume that when camphor is mixed with 1,8-cineole, its spreadability (i.e., the affinity) to the insects’ wax layer is increased, by lowering its surface tension compared to when it is individually administered (the actual difference in contact angles can be visually noticed, see Supplementary Fig. S1 online). Since human skin and insect integument share lipophilic properties, the penetration-enhancing effect of pharmaceutical drugs by 1,8-cineole to mammalian skin supports this finding. One difference might be the role of 1,8-cineole, which in the pharmaceutical area, is usually restricted to that of a penetration enhancer, not an active constituent, but for insecticidal activity, it needs to also be considered as an active principle.

**Figure 4:**
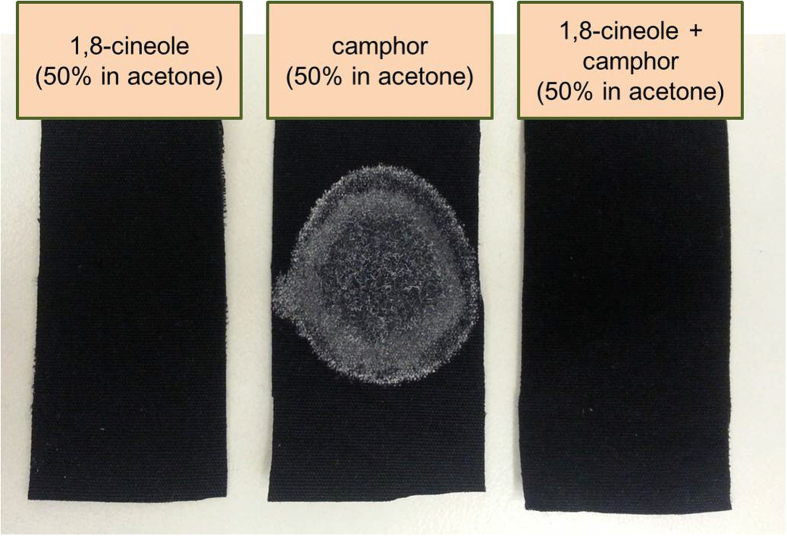
A comparison of the applied status of 1,8-cineole and camphor solutions on black cotton fabric. When 100 mg of camphor (50%, w/v in acetone) was applied on fabric, it was recrystallized making a white circle, whereas when the same amount of camphor was mixed with 1,8-cineole and applied (1,8-cineole : camphor = 63 : 37, 50%, w/v in acetone), there was no trace of solid crystals.

Further, 1,8-cineole may not only interact with the wax layer of insect integument but directly with camphor as well. Another possible hypothesis for increased penetration of camphor by 1,8-cineole might be through increased solubility. When 100 mg of camphor was dissolved in acetone and applied to black cotton fabric (50%, w/v), camphor recrystallized after the solvent evaporated (Fig. 4). When the same amount of camphor was applied on the fabric as a mixture with 1,8-cineole in acetone, no solid crystals were observed, indicating that the compounds can physically interact with each other, increasing the solubility of camphor, by changing it into a liquid phase. The liquefied camphor will have a higher mobility than the solid phase, and this can also contribute to the increased penetration rate. This increased solubility of camphor and its with increased penetration may also explain the synergistic effect of other combinations of monoterpene compounds. For example, in a previous report, the mixture of camphor and α-pinene showed synergistic toxicity^6^. Although α-pinene did not show any penetration-enhancing effect of drugs on human skin^41^, this may be a consequence of different characteristics of human skin and insect integument, but if α-pinene can somehow facilitate the solubility of camphor, then this insecticidal synergistic interaction can be explained in a similar fashion as well.

Although camphor showed higher toxicity than 1,8-cineole in certain other insects and arthropods such as the armyworm, *Pseudaletia unipuncta*^56^, and the food mite, *Tyrophagus putrescentiae*^57^, 1,8-cineole has long been considered as the active insecticidal constituent of rosemary oil against many arthropod species including the cabbage looper^6^,^56^ and stored-product insects^58^ because of its high concentration in the oil and higher individual bioactivity. In the present study, however, camphor showed better internal toxicity (via injection) with enhanced penetration, indicating its more significant contribution to overall toxicity.

Although our chromatographic investigations of penetration showed good correspondence with toxicity, a new technique that can trace penetration, and segregate potentially confounding enzymatic reactions would be useful. Since the introduced compounds can be readily detoxified by insects, what we observed might not represent the full amount that penetrated, but the final result of complex metabolic interactions at the specific time of sampling. To address this problem, it may be necessary to adapt an *in vitro* membrane model from the pharmaceutical field, although careful attention will be required to select appropriate materials and methods, which may be challenging. Because of the similarity in function of bio-membranes of many organisms, this finding could have potential to be applied in other areas of research as well. Moreover, since the compositions of plant essential oils can vary not only with environmental and cultivation conditions but also with methods of extraction^59^, deeper understanding of biological interactions among constituents will aid in the development of optimal production and uses of botanical insecticides in the field.

## Methods

### Insect maintenance

Eggs of an insecticide-susceptible strain of the cabbage looper, *Trichoplusia ni* were obtained from Great Lakes Forestry Centre’s Insect Production Service (Sault Ste. Marie, ON, Canada). The insect colony was reared on a pinto bean-based artificial diet in the insectary at the University of British Columbia, Vancouver, BC, kept at 22–25 °C and 16:8 h LD photoperiod.

### Toxicity contributions of rosemary oil constituents

The rosemary oil was obtained from Intarome Fragrance & Flavor Corporation (Norwood, NJ, USA; Lot no. 044931). To examine the contributions of the major constituents of rosemary oil to overall activity, 1,8-cineole (99%) and camphor (96%) individually and as a binary mixture, at the equivalent amounts in LD_95_ (or LC_95_) and LD_50_ (or LC_50_) doses of rosemary oil, as well as the mixture of the remaining major constituents of the oil (≥95% purity) were applied to 3rd instar larvae of the cabbage looper. Bioassays were conducted via topical administration or fumigation, with the same methods described elsewhere^6^. Briefly, for topical administration, each of ten larvae received test compounds dissolved in 1 μL of acetone (with acetone alone for the controls) using a syringe attached to a repeating micro dispenser. Larvae were transferred to 7 cm diameter Petri dishes, and provided with around 0.5 g fwt of diet. In the fumigant assay, ten larvae and a piece of diet (0.5 g) were placed into a 5 cm diameter Petri dish and covered with a lid with 70 holes, then the edges were sealed with Parafilm. To a filter paper, 50 μL of acetonic solution of the test compounds was applied (and acetone alone for controls), and dried for 30 sec to allow evaporation of the solvent. The filter paper was attached to a bottom of another Petri dish, and they were combined and sealed with Parafilm. Since the diameter of the holes was smaller than that of the larvae, the insects were unable to directly contact the filter paper, but were exposed to compounds in the vapor phase (see Supplementary Fig. S2 online). In a previous report, the LD_95_ and LD_50_ values of rosemary essential oil in the topical assay were determined as 446.7 and 215.8 μg/insect, respectively, and LC_95_ and LC_50_ values in the fumigation assay as 175.8 and 107.4 μg/mL air, respectively^6^. The equivalent amounts of individual 1,8-cineole (37.6%, in concentration) and camphor (20.2%), and also their mixture (57.8%) for each lethal dose of rosemary oil were applied, and all of the major constituents which were more than 1% in concentration were mixed and examined as well (with or without 1,8-cineole + camphor, see Supplementary Table S2 online). Mortality was recorded after 24 h. Test conditions were the same as those for insect maintenance above, and all tests were repeated three times.

To determine the interactions between the constituents, the actual mortalities were compared to expected mortalities based on the equation (1)
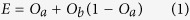
where *E* is expected toxicity and *O*_*a*_ and *O*_*b*_ are observed toxicities of individual compounds at given doses^60^. The interactions of the compounds can be designated synergistic, additive or antagonistic by determining χ^2^ values obtained from the equation (2)
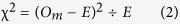
where *O*_*m*_ is the observed mortality of the mixture; χ^2^ with d.f. = 1 and α = 0.05 is 3.84. The interactions can be considered as synergistic when the χ^2^ values > 3.84 of the mixture and having greater mortality than the expected, and as antagonistic with smaller observed mortality than the expected, or as additive when χ^2^ values < 3.84.

### Sample preparations of 1,8-cineole and camphor for penetration analysis

Differences in penetration through the cuticle between individual compounds (1,8-cineole and camphor) and their binary mixture via topical application was monitored by GC-MS. Equivalent amounts of 1,8-cineole (98.7 μg) and camphor (56.9 μg) at the LD_50_ for the mixture (155.6 μg/insect)^6^ were topically applied individually or as a mixture to forty 3rd instar larvae of the cabbage looper. Treated larvae were transferred individually into 5 mL glass vials, and the caps were loosely fitted. After 10, 30, 60, and 180 min, thirty live larvae were selected and each larva was rinsed with 200 μL of n-hexane twice with gentle shaking for 30 sec. Rinses were pooled. Larvae were then pooled and ground using a tissue homogenizer for 1 min with 1 mL n-hexane. Five mL of n-hexane was added and sonicated for 30 sec, the supernatant was transferred into a glass vial, and sonication was repeated again with another 6 mL of n-hexane. The extracted and rinsed hexane solutions were kept sealed in a freezer overnight and analyzed.

### GC-MS analysis of 1,8-cineole and camphor uptake

Both rinsed and extracted solutions were analyzed by GC-MS in splitless mode using an Agilent 6890A series GC system (Agilent Technologies Canada Inc., Ottawa, ON, Canada) coupled to an Agilent 5973 Network MSD (70 eV). The injection volume was 200 μL, the oven temperature setup was 40 °C for 3 min, increasing at 25 °C/min to 300 °C then held for 5 min. Total run time was 18.4 min, and an HP-5ms column (30 m × 0.25 mm × 0.25 μm, Agilent) was selected with helium as carrier at 0.9 mL/min of flow. For quantification of compounds, five concentrations of each compound were used to prepare standard curves (see Supplementary Fig. S3 online), and 5 μg/mL of α-pinene was used as an internal standard. All analyses were done three times.

### Injection assay

To evaluate the effect of the cuticle as a barrier to penetration, an injection assay was conducted using 5th instar larvae of the cabbage looper. Ten larvae were placed into an ice-cold beaker for 5 min to slow their movement, and one μL of an acetonic solution of 1,8-cineole and camphor (or acetone alone for controls) was injected into the ventral body cavity close to the nerve cord using a microneedle (O.D. × I.D. = 0.47 × 0.21 mm) under a microscope (see Supplementary Fig. S4 online). Insects were transferred into 7 cm diameter Petri dishes, provided with 1 g of artificial diet. After 24 h, mortality was recorded and confirmed after 48 h. Seven doses were used to determine LD_50_ values. The LD_50_ values from injection were compared to those from topical administration against 3rd instar larvae from a previous study^6^ by using the equation (3)
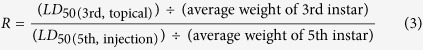
To calculate the average weight of 3rd instar larvae, ten larvae were grouped and weighed together with 5 replications (9.4 ± 1.6 mg/larva), and twenty 5th instar larvae were weighed individually (238.4 ± 27.6 mg/larva).

### Comparative toxicity in mixed application assay

To confirm the penetration enhancing effect of the mixture and its relationship to increased toxicity, and to examine whether injecting one compound can affect the other compound’s penetration rate, a mixed application assay was performed using 5th instar larvae. In this test, the compounds were applied as follows: (A) a mixture of 1,8-cineole and camphor was topically applied (64:37), (B) a mixture of 1,8-cineole and camphor was injected (mixture ratio was calculated based on the average penetration rate of each compound in the mixture’s application, 41:59, the calculations for each application can be found as Supplementary Table S3 online), (C and D) one compound was injected first and the other compound was topically applied (applied amount was determined based on the individual compound’s penetration rate), (E and F) an individual compound was either topically applied or injected. Mortalities resulting from each test (A to D) were compared to the sum of mortalities from individual application in the topical or injection assay (E and F). All the tests were repeated three times, and each test had ten larvae.

### Surface tension and contact angle measurement

The physical properties of rosemary oil and its major constituents were investigated by measuring surface tensions as well as their contact angles on a wax layer. Surface tensions of rosemary oil, 1,8-cineole, 1,8-cineole + camphor (63:37), and a full mixture minus 1,8-cineole were measured using a tensionmeter (K100, KRŰSS GmbH, Hamburg, Germany) by the Wilhelmy plate method. To measure the contact angle on a wax layer, beeswax specimens were prepared by dipping microscope slides into melted beeswax, and the specimens were air-dried overnight. Test solutions were prepared using acetone (50%, w/v), and 3 μL of solution was applied on the specimen and the contact angles were measured (DSA100, KRŰSS GmbH).

### Statistics

Statistical differences of mortalities from each bioassay were determined by one-way ANOVA with Tukey’s test post hoc, and probit analysis was used to calculate LD_50_ values using StatPlus 2009 (AnalystSoft, Alexandria, VA, USA). Values for penetration of compounds from individual and mixed applications within a time of observation were compared using Student’s t-test with the same software.

## Additional Information

**How to cite this article**: Tak, J.-H. and Isman, M. B. Enhanced cuticular penetration as the mechanism for synergy of insecticidal constituents of rosemary essential oil in *Trichoplusia ni. Sci. Rep*. **5**, 12690; doi: 10.1038/srep12690 (2015).

## Supplementary Material

Supplementary Information

## Figures and Tables

**Table 1:** Comparison of the insecticidal activities of 1,8-cineole and camphor in the topical and injection assays against 3rd and 5th instar larvae of *Trichoplusia ni*.

Compound	Topical (3rd instar)^a^		Injection (5th instar)		*R*^d^
	n^b^	LD_50_ μg/larva (95% CL^c^)	n^b^	LD_50_ μg/larva (95% CL^c^)	
1,8-cineole	360	229.6 (171.3–341.6)	210	374.3 (301.0–476.4)	15.6
camphor	240	471.3 (402.4–538.2)	210	147.0 (109.9–200.1)	81.3

^a^Data from previous report^6^.

^b^Number of insects used to determine LD_50_.

^c^Confidence limit.

^d^Toxicity ratio between injection assay and topical application assay.

**Table 2:** Surface tension and contact angle measurement.

	surface tension^a^ (mN/m ± s.d.)	contact angle^a^ (° ± s.d., 50% solution in acetone)
rosemary oil	29.8 ± 0.09 B	22.7 ± 1.7 B
1,8-cineole	29.2 ± 0.05 D	19.5 ± 1.6 C
camphor		58.3 ± 2.4 A
1,8-cineole + camphor^b^	30.0 ± 0.03 A	24.1 ± 1.0 B
(full mixture) – 1,8-cineole^c^	29.3 ± 0.07 C	23.9 ± 1.0 B

^a^Within each column means followed different letters are statistically different at *P* = 0.05 (Tukey’s test).

^b^The ratio of the mixture of 1,8-cineole + camphor was 63:37 (w:w), their natural ratio in rosemary oil.

^c^Full mixture is the mixture of all rosemary essential oil constituents occurring at more than 1% concentration in the oil.
