# Supplementary material for: Enhanced cuticular penetration as the mechanism for synergy of insecticidal constituents of rosemary essential oil in Trichoplusia ni
Source: Sci Rep. 2015 Jul 30;5:12690. doi: 10.1038/srep12690 (PMC4649899; doi:10.1038/srep12690)
Supplement: Supplementary Information [file srep12690-s1.pdf]

Supplementary Information of

Enhanced cuticular penetration as the mechanism for synergy of insecticidal constituents of rosemary essential oil in *Trichoplusia ni*

Jun-Hyung Tak and Murray B. Isman*

Faculty of Land and Food Systems, University of British Columbia, Vancouver, BC, Canada

* To whom correspondence should be addressed. E-mail: murray.isman@ubc.ca

Supplementary Table S1. GC-MS quantifications of 1,8-cineole and camphor in rinsed and extracted solutions at different observation times (n = 3)

	amount (µg/mL ± s.d.) from rinse		amount (µg/mL ± s.d.) from extract		Recovery (%)[a]	
	1,8-cineole	camphor	1,8-cineole	camphor	1,8-cineole	camphor
Individual[b]						
10 min	16.7 ± 0.2	34.7 ± 0.2	12.2 ± 0.3	4.0 ± 0.2	28.6	70.8
30 min	14.5 ± 0.8	47.6 ± 0.7	8.7 ± 0.1	9.1 ± 0.8	22.9	103.9
60 min	11.8 ± 0.1	21.9 ± 0.1	14.8 ± 0.3	13.1 ± 0.2	26.3	64.1
180 min	7.3 ± 0.2	10.4 ± 0.1	5.9 ± 0.1	8.5 ± 0.2	13.1	34.7
Mixture[c]						
10 min	30.2 ± 0.2	33.3 ± 0.5	12.7 ± 0.2	9.7 ± 0.4	42.4	78.8
30 min	27.9 ± 1.2	68.8 ± 2.3	15.3 ± 0.4	21.5 ± 0.7	42.7	165.2
60 min	14.8 ± 0.1	32.8 ± 0.3	16.6 ± 0.3	26.1 ± 0.6	31.0	107.9
180 min	14.2 ± 0.4	29.8 ± 0.8	13.3 ± 0.3	21.7 ± 0.4	27.2	94.4

[a]% based on the initially applied amount.

[b]Data from the individual application of each compound.

[c]Data when the two compounds were applied in mixture.

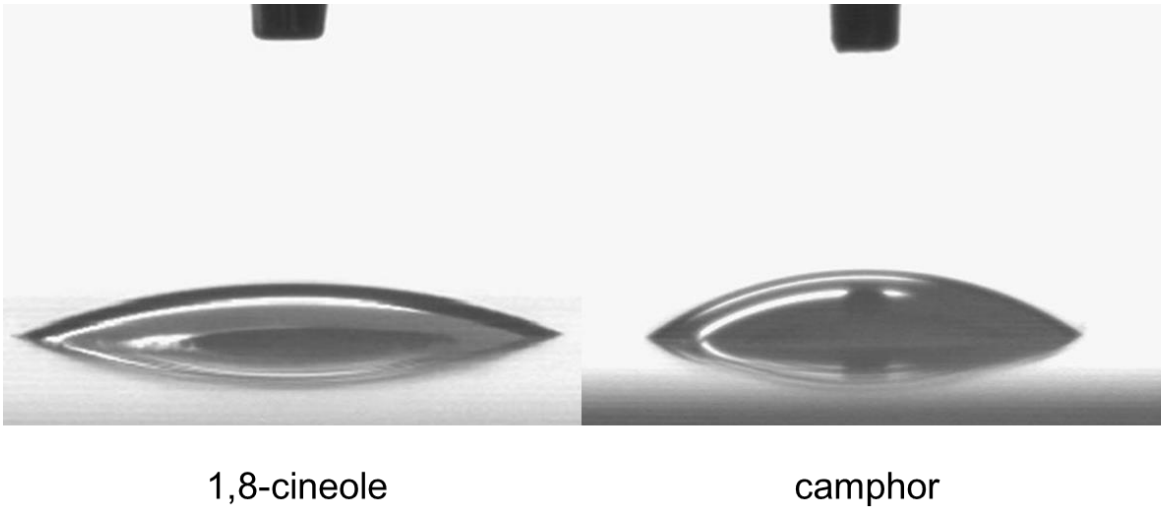

Supplementary Figure S1. Actual shapes of droplets of 1,8-cineole and camphor solutions (50%, w/v in acetone) from contact angle measurements. The acetonic solution of 1,8-cineole showed higher affinity (spreadability) to a beeswax layer than that of camphor.

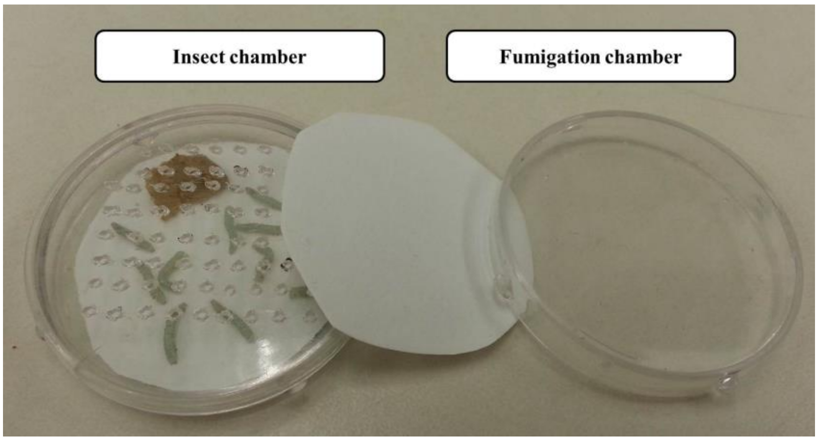

Supplementary Figure S2. Fumigant assay chamber system. Ten 3rd instar larvae of the cabbage looper, *Trichoplusia ni* were placed into a 5.5 cm diameter Petri dish with 0.5 g of artificial diet on a filter paper, and covered with an inner lid with 70 holes. A second filter paper, containing the test compounds was attached to the bottom of the fumigation chamber, and the two chambers were connected and sealed with parafilm.

Supplementary Table S2. The chemical composition of rosemary oil used in this study

constituent	% area[a]
1,8-cineole	37.6
(±)-camphor	20.2
(+)-α-pinene	15.7
camphene	7.9
(-)-β-pinene	4.3
p-cymene	2.5
(R)-(+)-limonene	2.1
L-bronyl acetate	2.1
α-terpineol	1.7
(-)-borneol	1.7
linalool	0.7
α-pinene oxide	0.5
iso-borneol	0.4
pinocarveol	0.3
α-fenchene	0.2
total	97.9

* data from Tak, J. H., Jovel, E. & Isman, M. B. Comparative and synergistic activity of *Rosmarinus officinalis* L. essential oil constituents against the larvae and an ovarian cell line of the cabbage looper, *Trichoplusia ni* (Lep., Noctuidae). *Pest Manag. Sci.* in press, DOI: 10.1002/ps.4010 (2015)

[a]All compounds at greater than 1% concentration were selected and mixed to make a ‘full mixture’ = artificial essential oil.

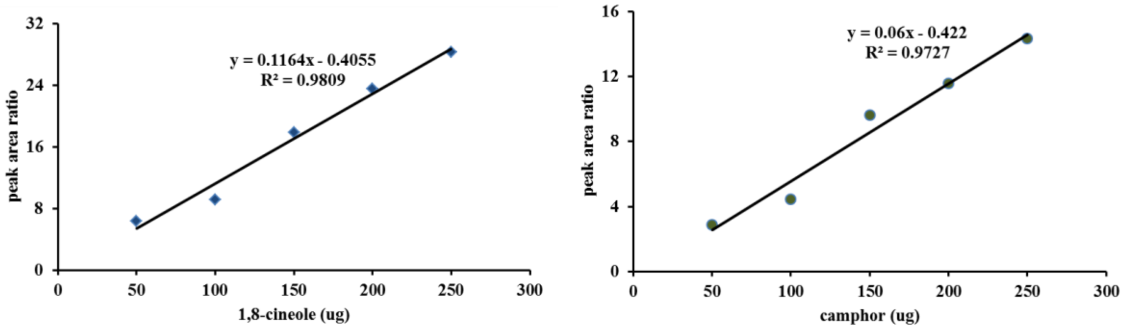

Supplementary Figure S3. Standard curves for GC-MS quantification. Five concentrations of each compound showed good linearity responses, and the ratio of the peak area of the compound and the inner standard was used to calculate the amount in the hexane solutions.

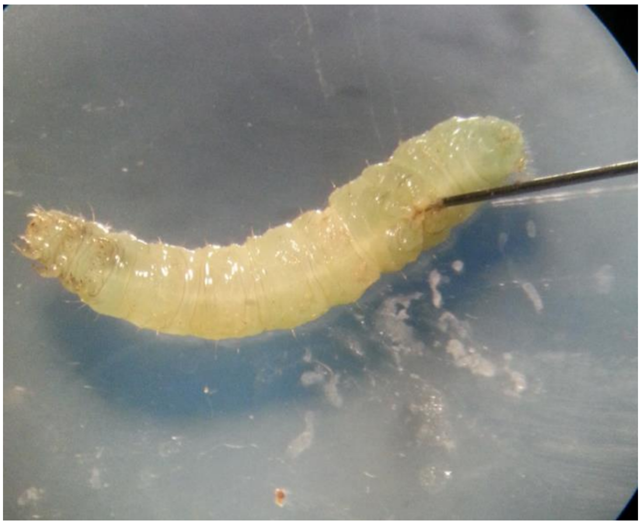

Supplementary Figure S4. Injection assay scheme. By using a microneedle, the test solution was injected into the ventral abdomen of 5th instar larvae of the cabbage looper, to the right of the nerve cord (the white line in the middle).

Supplementary Table S3. Test design of the mixed application assay with 5th instar larvae of the cabbage looper, *Trichoplusia ni*

	topical assay (ug/mL)		injection assay (ug/mL)		penetration (%)[a]		calculated amount of penetration (ug/mL)	
	1,8-cineole	camphor	1,8-cineole	camphor	1,8-cineole	camphor	1,8-cineole	camphor
A	717.8	414.4			14.3[b]	36.2[b]	102.6	150.0
B			102.6	150.0			102.6	150.0
C		943.4	102.6			15.9[c]	102.6	150.0
D	996.6			150.0	10.3[c]		102.6	150.0
E	996.6				10.3		102.6	
		943.4				15.9		150.0
F			102.6				102.6	
				150.0				150.0
A	358.9	207.2			14.3	36.2	51.3	75.0
B			51.3	75.0			51.3	75.0
C		471.7	51.3			15.9	51.3	75.0
D	498.3			75.0	10.3		51.3	75.0
E	498.3				10.3		51.3	
		471.7				15.9		75.0
F			51.3				51.3	
				75.0				75.0

[a]Average rate of penetration analyzed by GC-MS.

[b]In mixture, the average penetration of 1,8-cineole and camphor were 14.3 and 36.2%, respectively.

[c]When 1,8-cineole and camphor were applied individually, their penetrations were 10.3 and 15.9%, respectively.
